# Treatment toxicities and pathological response through the evolution of neoadjuvant regimens in early triple-negative breast cancer☆

**DOI:** 10.1016/j.esmorw.2025.100157

**Published:** 2025-06-03

**Authors:** E. Arnaud, P. Vaflard, L. Escalup, T. Ramtohul, D. Meziani, L. Thibault, R.-P. Desmaris, J.-G. Feron, J.-Y. Pierga, L. Cabel, F. Lerebours, D. Loirat, P. Cottu

**Affiliations:** 1 Department of Medical Oncology, Institut Curie, Paris, France; 2 Department of Pharmacy, Institut Curie, Paris, France; 3 Department of Radiology, Institut Curie, Paris, France; 4 Department of Pathology and Theranostic Medicine, Institut Curie, Paris, France; 5 Department of Surgery, Institut Curie, Paris, France; 6 Department of Université Paris Cité, Paris, France

**Keywords:** early-stage TNBC, neoadjuvant chemoimmunotherapy, pathological response, adverse events, real-world data

## Abstract

**Background:**

Pembrolizumab-based neoadjuvant chemoimmunotherapy (CIT) is the new standard of care for high-risk early triple-negative breast cancer (eTNBC). The addition of multiple cytotoxic drugs can lead to cumulative toxicities. We report the evolution of chemotherapy (CT) practice and its impact on completion and effectiveness of subsequent regimens in a real-world setting.

**Patients and methods:**

We conducted an ambispective, observational study of patients with eTNBC at a comprehensive cancer centre between February 2019 and February 2024. All data were extracted from electronic health records and manually curated.

**Results:**

Of the 366 patients enrolled, 247 received neoadjuvant CT and 119 received CIT. Grade 3/4 toxicities were more common in the CIT group (78.2% versus 58.6%, *P* < 0.001). The most common adverse events in both groups were haematological, gastrointestinal and general health deterioration [e.g. moving from Eastern Cooperative Oncology Group performance status (ECOG PS) 0-1 to ECOG PS 2-4]. In the CIT group, 56.8% of patients experienced at least one immune-related adverse event, 11.3% of which were grade 3/4. Toxicities led to a significantly higher rate of dose reductions (48.7% versus 25.0%, *P* < 0.0001), treatment delays (65.2% versus 27.5%, *P* < 0.0001) or permanent discontinuation (31.1% versus 16.0%, *P* < 0.001) in the CIT cohort. The pathological complete response (pCR) rate was significantly increased to 68.4% in the CIT group versus 51.9% in the CT group (*P* < 0.01). No effect of toxicities or dose adjustments on the pCR rate was observed in either group.

**Conclusions:**

Escalation of neoadjuvant CIT in TNBC increases toxicity and leads to significant dose reductions without affecting the improvement in pCR rate.

## Highlights

• This is an ambispective real-world study of neoadjuvant therapy in TNBC.
• The addition of carboplatin and pembrolizumab results in high toxicity burden.
• CT dose intensity is reduced when carboplatin and pembrolizumab are added.
• The pCR rate is not affected by dose intensity reduction.

## Introduction

With ∼2.3 million new cases in 2022, breast cancer is the most commonly diagnosed cancer in women worldwide.^1^ Triple-negative breast cancer (TNBC) accounts for ∼15% of all breast cancers^2^^,^^3^ and is the most aggressive subtype, with a higher risk of recurrence and mortality than other subtypes.^4^, ^5^, ^6^ The neoadjuvant setting is the gold standard for the treatment of early TNBC (eTNBC). Cytotoxic chemotherapy (CT) based on anthracyclines and taxanes, then carboplatin, has long been the backbone of standard treatment, improving early outcomes including pathological complete response (pCR), breast conservation and event-free survival (EFS).^7^ The achievement of pCR, which refers to the absence of residual invasive cancer cells in the breast and lymph nodes, is associated with longer EFS and overall survival (OS).^8^^,^^9^ In addition, the assessment of pathological response at the time of definitive surgery allows for individualisation of adjuvant treatment.

Recently, the phase III KEYNOTE-522 (KN-522) trial changed practice by showing that neoadjuvant pembrolizumab—a monoclonal antibody directed against programmed cell death protein 1—in combination with CT resulted in a statistically significant and clinically meaningful increase in pCR, EFS and OS.^10^^,^^11^ Although outcome data did not show an overall decrease in quality of life with the addition of pembrolizumab to CT,^12^ 76.8% of patients experienced treatment-related adverse events (TrAEs) of grade ≥3, leading to discontinuation of the study regimen in 27.7% of patients in the pembrolizumab–CT arm.^10^

The first objective of the present report was to describe how the rapid evolution of practice in the neoadjuvant setting for patients with eTNBC has affected adverse events and treatment management in a real-world setting in a comprehensive cancer centre. Secondly, we analysed clinical outcomes (pathological response and survival) according to treatment and for the entire cohort.

## Patients and methods

### Study design and population

We developed an ambispective cohort at a comprehensive cancer centre. Patients were included if they were at least 18 years of age and had centrally confirmed TNBC, defined as the absence of estrogen receptor (ER, expressed in <10% of tumour cells), progesterone receptor (PgR, expressed in <10% of tumour cells) and human epidermal growth factor receptor 2 (HER2) overexpression (score 0 or 1+ or 2+/FISH negative) based on biopsy samples. Patients were newly diagnosed, previously untreated and had non-metastatic disease as defined by clinical and imaging assessment (T1c N1-N2 or T2-4 N0-2 disease) according to the American Joint Committee on Cancer (AJCC) staging criteria (eighth edition). All patients included had received at least one CT regimen (CT cohort) or CT in combination with pembrolizumab [chemoimmunotherapy (CIT) cohort]. According to local guidelines, all patients were to be clinically assessed at each course. Magnetic resonance imaging (MRI) of the breast was scheduled at baseline, mid-term and preoperatively. After surgery, a 6-monthly clinical assessment with annual breast imaging was planned.

### Data collection

Data were collected using the centre’s electronic health record (EHR) system, including electronic pharmacy records. Data from the CIT cohort were collected consecutively and prospectively between July 2023 and February 2024, and data of the CT cohort were collected consecutively and ambispectively between June 2022 and February 2024. The list of patients was provided by the centre’s pharmacist using the keywords ‘neoadjuvant treatment’, ‘breast cancer’, ‘no anti-HER2’ and ‘no pembrolizumab’. A manual sorting process was then carried out, excluding patients who did not have TNBC. We did not exclude stage I tumours as some guidelines recommend neoadjuvant treatment for stage Ic cancers.^13^ Patients were enrolled backwards in a consecutive manner, achieving a ratio of ∼2 : 1 to the CIT cohort, with the earliest patients enrolled in February 2019. Overall, the study period spanned from February 2019 to February 2024. None of the patients in the CIT group received CT alone. We collected demographic data {sex, age, body mass index, Eastern Cooperative Oncology Group performance status (ECOG PS), menopausal status, comorbidities [tobacco, hypertension, diabetes, cardiopulmonary history (myocardial infarction, stroke, obstructive sleep apnoea syndrome), cancer history (breast cancer or other), autoimmune history for the CIT cohort], germline mutation}, tumour data [T, N, AJCC stage, histological subtype, grade, ER expression, PgR expression, HER2 status, Ki67, androgen receptor expression, tumour-infiltrating lymphocyte (TIL) count], treatment received, treatment progression, imaging studies, surgical management, pathological response after neoadjuvant treatment according to the residual cancer burden (RCB) grading system,^14^ adjuvant treatments and events (local progression as defined by clinical and/or imaging progression during neoadjuvant therapy, local relapse, contralateral relapse, metastases, other invasive cancer, death from any cause). Adverse events were manually extracted and graded from each individual EHR. Adverse events that may be related to either CT or pembrolizumab are reported along with non-immune-related adverse events (IrAEs).

### Ethical statement

Data collection was approved by our Institutional Review Board (IRB #DATA220277) and the study was conducted in accordance with the Declaration of Helsinki. Written informed consent was waived in accordance with current French regulations.

### Primary and secondary endpoints

The primary endpoint was an exploratory comparison of adverse events between the two cohorts. Adverse events were monitored throughout the neoadjuvant phase at each hospital site in all patients receiving at least one drug and were described according to the National Cancer Institute’s Common Terminology Criteria for Adverse Events version 5.0 (CTCAE v5). The adverse event grade recorded was the worst grade observed for each adverse event. IrAEs were monitored identically in the CIT cohort.

Secondary endpoints included RECIST objective response (by MRI), pCR rate according to the RCB definition^14^ in each cohort, EFS, distant metastasis-free survival (DMFS) and OS in the entire cohort. We did not compare the survival outcomes between the two cohorts.

EFS was defined as the time from diagnosis to progression during neoadjuvant therapy, first invasive locoregional or distant recurrence, treatment-related death or breast cancer-related death. DMFS was defined as the time from diagnosis to metastatic relapse. OS was defined as the time from diagnosis to death from any cause.

### Management of adverse events

Dose reduction was defined as a reduction in the dose of any drug. Treatment interruption was defined as a temporary postponement of any drug resulting in a delay in completion of treatment. Treatment discontinuation was defined as permanent discontinuation of any drug. Patients may have experienced more than one event. All events were considered and recorded. Relative dose intensity (RDI) was calculated for each molecule and defined as the ratio between the actual dose intensity received [in mg/m^2^/week or area under the concentration–time curve (AUC)/week] and the theoretical dose intensity planned (in mg/m^2^/week or AUC/week).

### Statistical analyses

Conventional statistics were used to describe continuous and categorical variables. Multiple logistic regression was used to assess the effect of multiple variables on the achievement of pCR. All variables potentially associated with pathological response were used. We tested for collinearity of categorical variables using Cramer’s V statistic and the goodness of fit of the model using the Hosmer–Lemeshow test. Survival curves were assessed using the Kaplan–Meier method, and groups were compared using the log-rank test. Analyses were carried out using MedCalc 20.2 (MedCalc Software Ltd, Ostend, Belgium; https://www.medcalc.org; 2022) and Graphpad Prism 10.1.2 (GraphPad Software, Boston, MA; www.graphpad.com) (324).

## Results

### Population and disease characteristics

Between February 2019 and February 2024, data were collected on 366 patients, including 247 patients in the CT cohort and 119 patients in the CIT cohort. In the CT cohort, 3 patients were lost to follow-up; 243 patients underwent surgery (2 lost to follow-up, 2 inoperable). Of the 119 patients included in the CIT cohort, 4 patients were lost to follow-up; 114 patients had data available for the effectiveness study (3 lost to follow-up, 2 inoperable). All these data are summarised in the flowchart (Supplementary Figure S1, available at https://doi.org/10.1016/j.esmorw.2025.100157). All patients were women. The median age at diagnosis was 47 years (range 39-57 years). The clinical features of the two cohorts were generally similar (Table 1). Nineteen percent and 15% of patients with available results in the CT and CIT cohorts, respectively, had a germline mutation. The most common mutation was *BRCA1* (70.2% in the CT cohort and 66.7% in the CIT cohort), followed by *BRCA2*.

**Table 1:** Demographic and disease characteristics at baseline

	Chemotherapy *n* = 247	Chemoimmunotherapy *n* = 119	*P* value^a^
Demographic characteristics			
Women, *n* (%)	247 (100)	119 (100)	
Age, years			
Median (range)	47 (39-57)	47 (39-55)	0.47
<40	63 (25.5)	33 (27.7)	0.65
≥40	184 (74.5)	86 (72.3)	
BMI, median (range), kg/m^2^	24.2 (22-27.7)	24.5 (21.6-28.5)	
ECOG PS score, *n* (%)			0.48
0	242 (98.0)	115 (96.6)	
1	5 (2.0)	4 (3.4)	
Menopausal status, *n* (%)			0.81
Premenopausal	146 (59.1)	66 (55.5)	
Postmenopausal	96 (38.9)	41 (34.5)	
Unknown	5 (2.0)	12 (10.0)	
Comorbidity, *n* (%)	101 (40.9)	37 (31.1)	0.07
Tobacco	43 (17.4)	16 (13.4)	0.37
High blood pressure	33 (13.4)	18 (15.1)	0.63
Diabetes	11 (4.5)	4 (3.4)	0.78
Cardiopulmonary history^b^	4 (1.6)	4 (3.4)	0.28
Other prior cancer	4 (1.6)^c^	5 (4.2)^d^	0.16
Autoimmune disease	Unknown	7 (5.9)	
Germline mutation	47 (24.5)^e^	18 (17.6)^e^	0.19
*BRCA1*	33 (70.2)	12 (66.7)	
*BRCA2*	8 (17.0)	3 (16.7)	
*PALB2*	2 (4.3)	1 (5.5)	
*RAD51*	4 (8.5)	2 (11.1)	
Wild type	145 (58.7)	84 (70.6)	
Untested	55 (22.3)	17 (14.3)	
Disease characteristic			
Primary tumour classification, *n* (%)			0.03 T1 versus T2+ <0.0001 T1-T2 versus T3-T4
T1	43 (17.4)	10 (8.4)	
T2	174 (70.4)	73 (61.3)	
T3	15 (6.1)	23 (19.3)	
T4a-c	5 (2.0)	7 (5.8)	
T4d	9 (3.6)	6 (5.0)	
Missing data	1 (0.4)	—	
Nodal involvement, *n* (%)			
N0	94 (38.1)	28 (23.5)	<0.01 N0 versus N+
N1	118 (47.8)	72 (60.5)	
N2	24 (9.7)	10 (8.4)	
N3	11 (4.5)	9 (7.5)	
AJCC disease stage, *n* (%)^f^			<0.01 I-IIA versus IIB-III <0.01 I-II versus III <0.01 IIA versus IIB-III
I	27 (10.9)	—	
IIA	80 (32.4)	32 (27.0)	
IIB	86 (34.8)	43 (36.1)	
IIIA	32 (13.0)	24 (20.2)	
IIIB	11 (4.5)	11 (9.2)	
IIIC	11 (4.5)	9 (7.6)	
Pathological subtype, *n* (%)			0.56
NST	226 (91.5)	107 (89.9)	
Lobular	2 (0.8)	1 (0.8)	
Other	18 (7.3)^g^	11 (9.2)^h^	
Missing data	1 (0.4)	—	
Tumour grade, *n* (%)			
1	1 (0.4)	—	0.88
2	41 (16.6)	19 (16.0)	Grade 2 versus 3
3	199 (80.6)	100 (84.0)	
Missing data	6 (2.4)		
Estrogen receptor expression, *n* (%)			0.03
<1%	216 (87.4)	94 (79.0)	
1%-9 %	30 (12.1)	25 (21.0)	
Missing data	1 (0.4)	—	
Progesterone receptor expression, *n* (%)			0.65
<1%	229 (92.7)	113 (95.0)	
1%-9 %	17 (6.9)	6 (5.0)	
Missing data	1 (0.4)	—	
HER2 status score, *n* (%)			0.68
0	142 (57.5)	66 (55.5)	
1+	72 (29.1)	31 (26.1)	
2+/FISH negative	32 (13.0)	22 (18.5)	
Missing data	1 (0.4)	—	
Ki67 expression, median (range), %	70 (50-80)	68 (50-80)	0.75
Ki67 < 30%	21 (8.5)	11 (9.2)	
Ki67 ≥ 30%	226 (91.5)	108 (90.8)	0.81
Androgen receptor expression, *n* (%)			0.97
<10%	151 (61.1)	54 (45.4)	
≥10%	58 (23.5)	21 (17.6)	
Missing data	38 (15.4)	44 (37.0)	
TILs			0.27
<30%	161 (65.1)	68 (57.1)	
≥30%	84 (34.0)	45 (37.8)	
Missing data	2 (0.8)	6 (5.0)	
Neoadjuvant treatment regimens			
A/EC q2w → P	167 (67.9)	—	
A/EC q3w → Tx	32 (13.0)^i^	—	
Any regimen + carboplatin	47 (19.1)^j^	119 (100)	
A/EC qw2 → P + Cb	23 (9.3)	—	
A/EC qw3 → P + Cb	6 (2.4)	1 (0.8)	
P + Cb → A/EC q2w	15 (6.1)	4 (3.4)	
P + Cb → A/EC q3w	2 (0.8)	109 (91.6)	
Cb → P	1 (0.4)	1 (0.8)	
Pembrolizumab	—	119 (100)	

A, doxorubicin; AJCC, American Joint Committee on Cancer; BMI, body mass index; C, cyclophosphamide; Cb, carboplatin; E, epirubicin; ECOG PS, Eastern Cooperative Oncology Group performance status; HER2, human epidermal growth factor receptor type 2; MRI, magnetic resonance imaging; NST, no special type; P, paclitaxel; q2w, every 2 weeks (high-dose pattern); q3w, every 3 weeks; TIL, tumour-infiltrating Lymphocyte; Tx, taxanes.

a Chi-square test, Fisher’s exact test, one-way analysis of variance.

b Myocardial infarction, stroke, obstructive sleep apnoea syndrome.

c Two Hodgkin lymphoma, two breast carcinomas.

d One Hodgkin lymphoma, three breast carcinomas, one Paget disease.

e Percentage on tested patients.

f Clinical AJCC stage according to T and N defined on clinical examination and MRI at diagnosis.

g Other histological subtypes in the chemotherapy cohort: eight metaplastic, four apocrine, three epidermoid, one micropapillary, one pleomorphic, one with lymphoid stroma.

h Other histological subtypes in the chemoimmunotherapy cohort: five metaplastic, three apocrine, one medullary, one basaloid, one oncocytic.

i 30 including paclitaxel, 2 including docetaxel.

j Percentage on non-missing data.

The most common histological tumour subtype in both cohorts was non-specific type (∼90% in both cohorts). Most patients had a T2 tumour (70.4% in the CT cohort, 61.3% in the CIT cohort) and lymph node involvement (62.0% in the CT cohort, 76.4% in the CIT cohort). As expected, since pembrolizumab is not approved in stage I tumours, patients in the CIT cohort had more severe disease overall (78.1% stage I-II in the CT cohort versus 63.1% in the CIT cohort, *P* < 0.01). We also observed an imbalance in the proportion of patients with low ER expression (1%-9%) (12.1% versus 21.0% in the CT and CIT cohorts, respectively, *P* = 0.03).

### Neoadjuvant treatments

In the CT cohort, patients received neoadjuvant CT consisting of anthracyclines (either epirubicin at 90 mg/m^2^ or doxorubicin at 60 mg/m^2^) combined with cyclophosphamide (600 mg/m^2^), administered every 2 weeks (dose-dense schedule) or every 3 weeks at the discretion of the physician, for four cycles. This was followed by taxanes, either paclitaxel (80 mg/m^2^ weekly) or docetaxel (100 mg/m^2^ every 3 weeks), for four cycles. With the introduction of carboplatin in the neoadjuvant treatment of eTNBC, 19% of patients additionally received carboplatin with taxanes after completing anthracyclines and cyclophosphamide, with dosing based on the AUC at the discretion of the physician. Further details are provided in Table 1. In the CIT cohort, patients received a neoadjuvant regimen consisting of four cycles of pembrolizumab (200 mg) every 3 weeks in combination with weekly paclitaxel (80 mg/m^2^) and carboplatin (dosing based on AUC as per the physician choice, details in Supplementary Table S1, available at https://doi.org/10.1016/j.esmorw.2025.100157). This was followed by four cycles of pembrolizumab (200 mg) in combination with doxorubicin (60 mg/m^2^) and cyclophosphamide (600 mg/m^2^), administered every 3 weeks.

### Non-immune-related adverse events

Almost all patients in both cohorts experienced at least one TrAE, with a rate of 99.2% in the CT cohort and 100% in the CIT cohort (Table 2). Common adverse events included anaemia, neutropenia, gastrointestinal problems (such as nausea, vomiting, diarrhoea or constipation) and general health deterioration (i.e. moving from ECOG PS 0-1 to ECOG PS 2-4, including anorexia, fatigue and weight loss). Overall, grade ≥3 adverse events were significantly more common in the CIT cohort than in the CT cohort (78.2% versus 58.6%, *P* < 0.001). Haematological toxicities were significantly more severe in the CIT cohort, with significantly higher rates of grade ≥3 anaemia (36.5% versus 7.4%, *P* < 0.001), thrombocytopenia (11.3% versus 2.0%, *P* < 0.001) and neutropenia (65.2% versus 55.3%, *P* < 0.001). Febrile neutropenia was also more common in the CIT group (25.2% versus 8.6%, *P* < 0.001). Red blood cell transfusions were required to treat haematological toxicities in 46 patients (40.7%) in the CIT cohort and 26 patients (10.7%) in the CT cohort (*P* < 0.001). Iron supplementation, mainly intravenous, was required more frequently in the CIT group (28.7% versus 13.5%, *P* < 0.001). In addition, platelet transfusions were required in six patients (5.2%) in the CIT cohort compared with none in the CT cohort. General health deterioration of any grade was significantly higher in the CIT group (92.2% versus 70.5%, *P* < 0.001), as were gastrointestinal toxicities (90.5% versus 78.7%, *P* < 0.01). Cardiotoxicity attributed to anthracyclines was also more common in the CIT cohort (4.3% versus 0.8%, *P* = 0.04). In the subset of CT patients who received carboplatin, the rate of grade ≥3 adverse events was numerically higher at 80.9%, particularly for haematological toxicities of grade ≥3 (12.8% for anaemia, 6.4% for thrombocytopenia and 76.6% for neutropenia) and general health deterioration of any grade (83.0%) (Supplementary Table S2, available at https://doi.org/10.1016/j.esmorw.2025.100157). There was one death due to coronavirus disease 2019 (COVID-19) in the CT cohort during the neoadjuvant phase, but no adverse events were reported in either cohort.

**Table 2:** Non-immune treatment-related adverse events

	Chemotherapy cohort *n* = 244			Chemoimmunotherapy cohort *n* = 115			*P* value
	Any grade *n* (%)	Grade 2 *n* (%)	Grade ≥ 3 *n* (%)	Any grade *n* (%)	Grade 2 *n* (%)	Grade ≥ 3 *n* (%)	
Any adverse event	242 (99.2)		143 (58.6)^1^	115 (100)		90 (78.2)^1^	<0.001^1^
Anaemia	225 (92.2)		18 (7.4)^2^	115 (100)		42 (36.5)^2^	<0.0001^2^
Thrombocytopenia	20 (8.2)^3^		5 (2.0)^4^	48 (41.7)^3^		13 (11.3)^4^	<0.000001^3^ <0.001^4^
Neutropenia	184 (75.4)		135 (55.3)^5^	99 (86.1)		75 (65.2)^5^	<0.001^5^
Febrile neutropenia	21 (8.6)			29 (25.2)			<0.00001
Peripheral neuropathy	158 (64.8)^6^	37 (15.2)	7 (2.9)	64 (55.7)^6^	12 (10.4)	2 (1.7)	0.09^6^
Skin reaction	44 (18.0)^7^	6 (2.5)	3 (1.2)	16 (13.9)^7^	4 (3.5)	1 (0.9)	0.37^7^
Cardiotoxicity	2 (0.8)^8^	1 (0.4)	—	5 (4.3)^8^	1 (0.9)	—	0.04^8^
General health deterioration	172 (70.5)^9^	51 (20.9)	2 (0.8)	106 (92.2)^9^	56 (48.7)	12 (10.4)	<0.0001^9^
Gastrointestinal toxicity	192 (78.7)^10^	68 (27.9)	5 (2.0)	105 (90.5)^10^	55 (47.8)	9 (7.8)	<0.01^10^
Death	1 (0.4)^a^			—			

^1, 2, 3, 4, 5, 6, 7, 8, 9, 10^The exponent relates the *P* values to corresponding values on the same line.

a Due to COVID-19 infection.

### Immune-related adverse events

Among the 115 patients in the CIT cohort with available safety data, 65 (56.5%) experienced at least one IrAE of any grade, with 11.3% of these events being grade ≥3. The most common IrAEs were hypothyroidism (20.0%), hyperthyroidism (17.4%) and troponin elevation (24.3%), of which 7.0% were diagnosed as myocarditis (confirmed by MRI according to the Lake Louise criteria and/or myocardial biopsy). Adrenal insufficiency occurred in 7.0% of patients. Detailed information on IrAEs is shown in Table 3.

**Table 3:** Immune-related adverse events in the chemoimmunotherapy cohort

	*n* = 115		
	Any grade *n* (%)	Grade 2 *n* (%)	Grade ≥ 3 *n* (%)
Any adverse event	65 (56.5)	33 (28.7)	13 (11.3)
Hypothyroidism	23 (20.0)	18 (15.7)	—
Hyperthyroidism	20 (17.4)	3 (2.6)	2 (1.7)
Skin reaction	5 (4.3)	—	2 (1.7)
Adrenal insufficiency	8 (7.0)	7 (6.1)	1 (0.9)
Pneumonitis	2 (1.7)	1 (0.9)	1 (0.9)
Hypophysitis	3 (2.6)	2 (1.7)	—
Nephropathy	1 (0.9)	—	1 (0.9)
Troponin elevation	28 (24.3)	28 (24.3)	—
Myocarditis	8 (7.0)		
Proven by myocardial biopsy	4 (3.5)		
Pericarditis	1 (0.9)	1 (0.9)	—
Liver enzyme elevation^a^	11 (9.6)	3 (2.6)	4 (3.5)
Pancreatitis	1 (0.9)	—	1 (0.9)
Arthritis	2 (1.7)	1 (0.9)	1 (0.9)

a Defined by elevated aspartate aminotransferase and/or alanine aminotransferase levels.

### Impact of toxicities on treatment course

**Figure 1:**
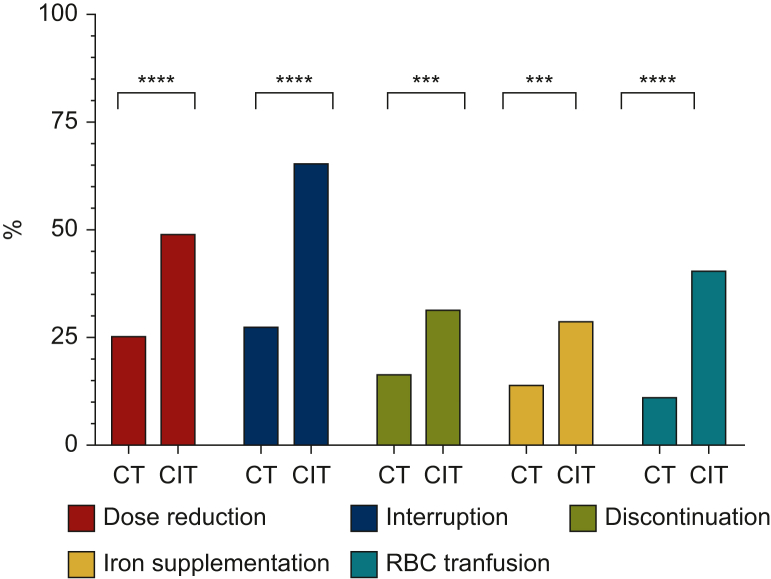
**Impact of toxicities on treatment course and haematological toxicity management according to neoadjuvant treatment.** Bar plot showing the proportion of dose adjustments and the requirements for haematological toxicity management. Dose reduction is defined as a reduction in the dose of any drug. Interruption is defined as transient postponement of any drug. Treatment discontinuation is defined as permanent interruption of any drug. CIT, chemoimmunotherapy; CT, chemotherapy; RBC, red blood cells. ∗∗∗*P* < 0.001; ∗∗∗∗*P* < 0.0001.

Adverse events led to a significantly higher rate of dose reductions of at least one drug in the CIT cohort (48.7% versus 25.0% in the CT cohort, *P* < 0.001). Treatment discontinuation of at least one drug due to TrAEs was also more common in the CIT group (65.2% versus 27.5% in the CT cohort, *P* < 0.001). Definitive discontinuation of at least one drug due to adverse events was significantly more frequent in the CIT cohort (31.1% versus 16.0% in the CT cohort, *P* < 0.001) (Figure 1). Details of treatment discontinuation for each individual drug are shown in Supplementary Figure S2, available at https://doi.org/10.1016/j.esmorw.2025.100157. In the CIT cohort, patients had a significantly lower RDI for anthracyclines (mean RDI 89% versus 99% in the CT cohort, *P* < 0.001) and cyclophosphamide (mean RDI 90% versus 99% in the CT cohort, *P* < 0.001). However, the RDI for carboplatin was significantly higher in the CIT cohort (mean RDI 92% versus 82% in the CT cohort, *P* < 0.001). There was no significant difference in RDI for taxanes (Supplementary Figure S2, available at https://doi.org/10.1016/j.esmorw.2025.100157).

### Tumour response

Mid-term imaging showed a higher objective tumour response in the CIT group (95.7% versus 88.8% in the CT cohort, *P* = 0.01). However, at preoperative imaging, there was no significant difference in response rate between the two groups (95.1% in CIT versus 92.7% in CT, *P* = 0.11). Rates of imaging progression during neoadjuvant treatment were similar between cohorts, with 3.4% in the CT group and 2.0% in the CIT group at preoperative imaging (Supplementary Table S3, available at https://doi.org/10.1016/j.esmorw.2025.100157). Partial mastectomy was the most common procedure in both cohorts (70.2% in CT and 64.0% in CIT), with sentinel node dissection more common in the CT group (62.6%) and axillary dissection more common in the CIT group (57.0%) (Supplementary Table S4, available at https://doi.org/10.1016/j.esmorw.2025.100157).

**Figure 2:**
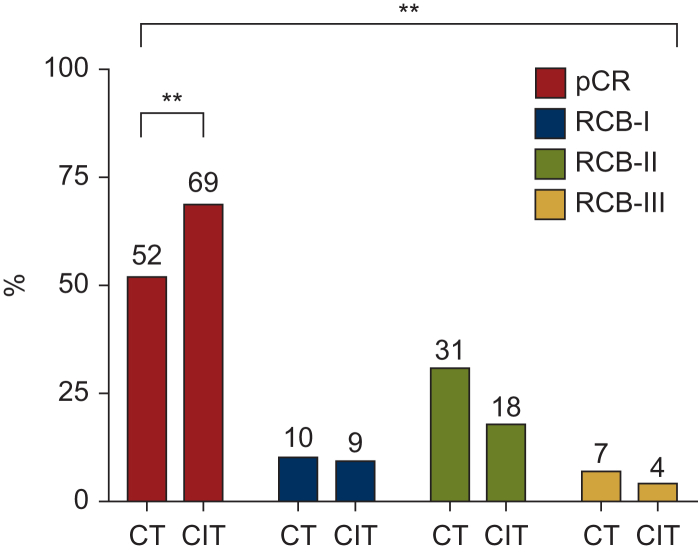
**Pathological response after neoadjuvant setting according to neoadjuvant treatment.** Bar plot showing pathological response according to the RCB system. CIT, chemoimmunotherapy; CT, chemotherapy; pCR, pathological complete response; RCB, residual cancer burden; RCB-0, no tumour residue or pCR; RCB-I, minimal tumour residue; RCB-II, moderate tumour residue; RCB-III, extensive tumour residue. The numbers indicate the actual percentages. ∗∗*P* < 0.01.

pCR was achieved in 51.9% of patients in the CT cohort and 68.4% in the CIT cohort (*P* < 0.01). Among patients without pCR, 10.3% and 8.8% were classified as RCB-I, 30.9% and 18.4% as RCB-II and 7.0% and 4.4% as RCB-III in the CT and CIT cohorts, respectively (overall *P* < 0.01, Figure 2). In an analysis of the relationship between pathological response and treatment toxicity, the occurrence of grade ≥3 toxicities did not affect pCR in either group. Most importantly, dose reductions, treatment delays or permanent treatment discontinuation did not affect pCR in both univariate and multivariate analyses (Supplementary Table S5, available at https://doi.org/10.1016/j.esmorw.2025.100157, and Table 4). Notably, pCR was independently associated with the addition of pembrolizumab and high tumour proliferation. The Cramer’s V statistics were <0.25, indicating a low to very low probability of collinearity between variables. The Hosmer–Lemeshow test was not significant (*P* = 0.50), indicating a reasonable fit of the model.

**Table 4:** Multivariate analysis of the impact of demographic factors, occurrence of toxicities or treatment interruption on pCR

	OR (95% CI)
Age, years	
≥40	1
<40	1.151 (0.6414-2.086)
AJCC disease stage	
III	1
II	1.385 (0.7536-2.553)
TILs, %	
<30	1
≥30	1.568 (0.9137-2.722)
Ki67, %	
<30	1
≥30	**8.720 (2.688-39.62)**
Androgen receptor expression	
Negative	1
Positive	1.637 (0.9153-2.985)
Treatment	
CT	1
CIT	**2.959 (1.537-5.933)**
Grade ≥3 AE	
Yes	1
No	1.141 (0.6394-2.047)
Dose reduction	
No	1
Yes	1.408 (0.7661-2.619)
Postponement of Tx	
No	1
Yes	0.89 (0.48-1.66)
Permanent discontinuation of Tx	
No	1
Yes	0.5306 (0.2632-1.055)

OR significantly greater than 1 are indicated in bold.

AJCC, American Joint Committee on Cancer; AE, adverse event; CI, confidence interval; CIT, chemoimmunotherapy; CT, chemotherapy; OR, odds ratio; TIL, tumour-infiltrating lymphocyte; Tx, taxanes.

### Adjuvant treatment and survival

Following surgery, 96.4% of patients in the CT cohort and 93.3% in the CIT cohort received radiotherapy according to local guidelines. Of those who achieved a pCR, 100% in the CT cohort and 17.9% in the CIT cohort received no adjuvant treatment, with 82.1% of CIT patients receiving adjuvant pembrolizumab (Supplementary Table S6, available at https://doi.org/10.1016/j.esmorw.2025.100157). Among patients who did not achieve pCR, 79.5% and 33.3% received capecitabine, 3.4% and 2.8% received olaparib and 10.3% and 38.9% received pembrolizumab in the CT and CIT cohorts, respectively. In addition, 23.1% of patients in the CIT group without pCR received adjuvant pembrolizumab. After a median follow-up of 38.7 months in the CT cohort and 18.9 months in the CIT cohort, 43 events (17.4%) were reported in the CT cohort and 9 events (7.6%) in the CIT cohort. The most common event in the CT cohort was distant recurrence after surgery (79.1%), while in the CIT cohort it was progression during neoadjuvant treatment (66.7%). Distant recurrence remained the predominant type of relapse in both cohorts (82.9% in CT and 55.6% in CIT), with details of metastatic sites shown in Supplementary Table S7, available at https://doi.org/10.1016/j.esmorw.2025.100157. During follow-up, 18 patients (7.3%) in the CT cohort and 1 patient (0.8%) in the CIT cohort died, primarily from breast cancer (88.9% in CT and 100% in CIT) (Supplementary Table S7, available at https://doi.org/10.1016/j.esmorw.2025.100157). Achieving a pCR after neoadjuvant treatment significantly improved EFS (*P* < 0.0001), DMFS (*P* < 0.0001) and OS (*P* < 0.001) in the entire cohort (Supplementary Figure S4, available at https://doi.org/10.1016/j.esmorw.2025.100157).

## Discussion

We report here an ambispective real-world study of patients with eTNBC and treated with neoadjuvant CT or neoadjuvant CIT. We observed a striking increase of grade 3/4 toxicities in patients in the CIT cohort, as compared with patients in the CT cohort. These toxicities led to a greater number of dose reductions, treatment delays or discontinuation of the neoadjuvant sequence in the CIT cohort for almost all cytotoxic drugs, making the routine management of this therapeutic sequence more complex than conventional care. The need for haematological support was significantly higher in the CIT cohort, probably due to the systematic addition of carboplatin to the cytotoxic regimen. As a result, the dose intensity of almost all cytotoxic drugs was reduced in the CIT cohort. Patients in the CIT cohort also experienced IrAEs. Despite these limitations in completing the neoadjuvant course, the pCR rate was higher in patients who received pembrolizumab. As expected, factors associated with pCR by multivariate logistic regression analysis were the addition of pembrolizumab and a high proliferation rate.^15^ It is too early to draw conclusions about long-term events. These results are remarkably consistent with those of the KN-522 study both in terms of pCR rate with pembrolizumab (68.4% in our cohort versus 64.8% in the KN-522 study) and in patients who did not receive pembrolizumab (52% in our cohort versus 51.2% in the KN-522 study).^10^ This was also the case for grade ≥3 toxicities (78.2% in our cohort versus 77.1% in the KN-522 study) and impact on treatment discontinuation (31.1% in our cohort versus 27.7% in the KN-522 study) (Supplementary Table S8, available at https://doi.org/10.1016/j.esmorw.2025.100157). These results are also consistent with those of several real-world data reports.^16^, ^17^, ^18^

Several real-world studies haves evaluated the impact of CIT toxicities in eTNBC. One early study, conducted in only 35 patients, showed that the occurrence of IrAE significantly correlated with pCR (72.2% versus 30.8%, *P* = 0.03). In addition, a lower pCR rate was observed in patients with early CT discontinuation.^19^ Other studies reported similar incidence of adverse events, whether immune related or not.^20^^,^^21^ One study also suggested that reduction in the RDI of CT was associated with lower pCR rates.^20^ We did not observe these associations in our study. The high toxicity burden of the KN-522 regimen, which we confirm in our large real-world cohort, together with the apparent lack of detrimental effect of reducing the CT dose intensity, calls into question the need to de-escalate the CT regimen. The ongoing randomised phase III SCARLET trial (NCT05929768) is comparing the effects of a shorter CIT without anthracyclines with usual CIT for the neoadjuvant treatment of eTNBC. In addition, some features of TNBC may be associated with a favourable prognosis and these patients may be candidates for de-escalation of treatment. In particular, TILs have a significant impact on tumour prognosis,^22^, ^23^, ^24^ particularly high levels of CD8+ cells and a high CD8/CD4 ratio, which have been reported to be associated with the achievement of pCR.^25^ Thus, several studies suggest that TIL quantification can be used to identify a subset of patients with eTNBC who achieve excellent outcomes even without systemic treatment.^24^ In the present series, a high TIL level (≥30%) did not reach a significant association with pCR (odds ratio 1.57, 95% confidence interval 0.91-2.72), but was confirmed in a separate report.^26^

The main strength of our study is that it is the first real-world comparative study between CT and CIT with such a large number of patients. Nevertheless, we acknowledge certain limitations. This is a partially retrospective study with inherent biases due to the recording of adverse events through EHRs. We observed some imbalance in tumour stage between the two cohorts, which limits the interpretation of the pCR results. However, as the clinical characteristics were very similar, it is unlikely that these differences influenced the toxicity results. Furthermore, interestingly, our results tend to confirm that despite a higher tumour stage and toxicity burden in the CIT cohort, the KN-522 regimen definitely provides better pCR results. However, no formal matching was carried out between the two cohorts to ensure a robust comparison. In the CT arm, only 19% of patients received carboplatin, which biased the assessment of adverse events. However, discontinuation of carboplatin was specifically analysed and appeared to be more common in the CIT arm. We recognise that the survival analyses are exploratory and limited by the short follow-up and small number of events. In addition, the study was limited to a single expert centre. The impact of toxicities on patients’ quality of life was not assessed. Although the incidence of toxicities increased in patients receiving immunotherapy, this was not found to affect quality of life.^12^ However, it would be beneficial to evaluate this further in real-life settings, with a particular focus on long-term toxicities, especially those related to the immune system. This would allow a more comprehensive assessment of the impact of these toxicities on quality of life, including the need for longer-term monitoring of these toxicities.

### Conclusion

In summary, advances in neoadjuvant therapy for eTNBC have improved treatment efficacy and long-term outcomes. The addition of drugs to an existing CT regimen is responsible for an increase in toxicities, which can affect the completion of the therapeutic sequence. However, this does not appear to affect the improvement in the pCR rate. Further research is needed to propose a personalised treatment for each patient that aims to achieve pCR while limiting the occurrence of side-effects.
