# Supplementary material for: Treatment toxicities and pathological response through the evolution of neoadjuvant regimens in early triple-negative breast cancer
Source: ESMO Real World Data Digit Oncol. 2025 Jun 3;9:100157. doi: 10.1016/j.esmorw.2025.100157 (PMC12836798; doi:10.1016/j.esmorw.2025.100157)

**Supplementary Appendix**

| **Supplementary Table 1** – Details of Carboplatin dose based on an area under the concentration-time curve according to physician's choice in the CIT cohort. | |
| --- | --- |
| **Carboplatin dose** | **N = 119** |
| AUC5 (qw3) | 104 (89.7%) |
| AUC4 (qw3) | 2 (1.7%) |
| AUC2 (qw1) | 1 (0.9%) |
| AUC1.5 (qw1) | 9 (7.8%) |
| Missing data | 3 (2.6%) |
| *AUC: Area Under the Curve ; CIT : chemo-immunotherapy ; qw1 : every week ; qw3 : every 3 weeks.* | |

| **Supplementary Table 2** - Details of non-immune-related adverse events according to treatment regimen. | | | | |
| --- | --- | --- | --- | --- |
|  | **CT**  **N = 244** | **CT without Cb**  **N = 197** | **CT with Cb**  **N = 47** | **CIT**  **N = 115** |
| **Any adverse event** | 242 (99.2%) | 195 (99.0%) | 47 (100%) | 115 (100%) |
| Grade ≥ 3 | 143 (58.6%) | 105 (53.3%) | 38 (80.9%) | 90 (78.2%) |
| **Anemia – any grade** | 225 (92.2%) | 178 (90.4%) | 47 (100%) | 115 (100%) |
| Grade ≥ 3 | 18 (7.4%) | 12 (6.1%) | 6 (12.8%) | 42 (36.5%) |
| **Thrombocytopenia – any grade** | 20 (8.2%) | 5 (2.5%) | 15 (31.9%) | 48 (41.7%) |
| Grade ≥ 3 | 5 (2.0%) | 2 (1.0%) | 3 (6.4%) | 13 (11.3%) |
| **Neutropenia – any grade** | 184 (75.4%) | 141 (71.6%) | 43 (91.5%) | 99 (86.1%) |
| Grade ≥ 3 | 135 (55.3%) | 99 (50.3%) | 36 (76.6%) | 75 (65.2%) |
| **Febrile neutropenia** | 21 (8.6%) | 16 (8.1%) | 5 (10.6%) | 29 (25.2%) |
| **Peripheral neuropathy – any grade** | 158 (64.8%) | 133 (67.5%) | 25 (53.2%) | 64 (55.7%) |
| Grade 2 | 37 (15.2%) | 33 (16.8%) | 4 (8.5%) | 12 (10.4%) |
| Grade ≥ 3 | 7 (2.9%) | 7 (3.6%) | - | 2 (1.7%) |
| **Skin reaction – any grade** | 44 (18.0%) | 36 (18.3%) | 8 (17.0%) | 16 (13.9%) |
| Grade 2 | 6 (2.5%) | 5 (2.5%) | 1 (2.1%) | 4 (3.5%) |
| Grade ≥ 3 | 3 (1.2%) | 3 (1.5%) | - | 1 (0.9%) |
| **Cardiotoxicity – any grade** | 2 (0.8%) | 2 (1.0%) | - | 5 (4.3%) |
| Grade 2 | 1 (0.4%) | 1 (0.5%) | - | 1 (0.9%) |
| Grade ≥ 3 | - |  | - | - |
| **General health deterioration – any grade** | 172 (70.5%) | 133 (67.5%) | 39 (83.0%) | 106 (92.2%) |
| Grade 2 | 51 (20.9%) | 40 (20.3%) | 11 (23.4%) | 56 (48.7%) |
| Grade ≥ 3 | 2 (0.8%) | 1 (0.5%) | 1 (2.1%) | 12 (10.4%) |
| **Gastrointestinal toxicity – any grade** | 192 (78.7%) | 154 (78.2%) | 38 (80.9%) | 105 (90.5%) |
| Grade 2 | 68 (27.9%) | 54 (27.4%) | 14 (29.8%) | 55 (47.8%) |
| Grade ≥ 3 | 5 (2.0%) | 3 (1.5%) | 2 (4.3%) | 9 (7.8%) |
| **Death** | 1 (0.4%)^$^ | 1 (0.5%)^$^ | - | - |
| *^$^Due to Covid-19 infection.*  *Cb : Carboplatin ; CIT : chemoimmunotherapy ; CT : chemotherapy.* | | | | |

| **Supplementary Table 3** - Tumor response on imaging (MRI) through neoadjuvant treatment and pre-operatively. | | | |
| --- | --- | --- | --- |
|  | **CT**  **N = 247** | **CIT**  **N = 119** |  |
| **Mid-term imaging** |  |  |  |
| Objective tumor response  Without Stade I (N = 214)  Partial response  Without Stade I  Complete response  Without Stade I  Stability  Progression  Not realized  Missing data | 214 (88.8%)  189 (88.3%)  200 (93.5%)  176 (82.2%)  14 (6.5%)  13 (6.1%)  24 (10.0%)  3 (1.2%)  4 (1.6%)  2 (0.8%) | 90 (95.7%)  -  72 (80.0%)  -  18 (20.0%)  -  3 (3.2%)  1 (1.1%)  22 (18.5%)  3 (2.5%) | 0.011 |
| **Preoperative imaging** |  |  |  |
| Objective tumor response  Without Stade I (N = 208)  Partial response  Without Stade I  Complete response  Without Stade I  Stability  Progression  Not realized  Missing data | 217 (92.7%)  192 (92.3%)  136 (62.7%)  124 (64.6%)  81 (37.3%)  68 (35.4%)  9 (3.8%)  8 (3.4%)  12 (4.9%)  1 (0.4%) | 97 (95.1%)  -  61 (62.9%)  -  36 (37.1%)  -  3 (2.9%)  2 (2.0%)  14 (11.8%)  3 (2.5%) | 0.11 |
| *CIT : chemoimmunotherapy ; CT : chemotherapy.* | | | |

| **Supplementary Table 4** - Details of surgical management after neoadjuvant treatment. | | |
| --- | --- | --- |
|  | **CT** | **CIT** |
| **Breast surgery**  Breast conservation  Mastectomy | N = 242*  170 (70.2%)  72 (29.6%) | N = 114^#^  73 (64.0%)  41 (36.0%) |
| **Lymph node surgery**  Sentinel lymph node evaluation  Axillary dissection | N = 243^£^  152 (62.6%)  91 (37.4%) | N = 114  49 (43.0%)  65 (57.0%) |
| *CIT : chemoimmunotherapy ; CT : chemotherapy.*  **2 surgeries cancelled for progression, 1 death during neoadjuvant treatment due to Covid-19 infection, 1 lost to follow-up, 1 underwent axillary dissection without breast procedure.*  *^£^ 2 surgeries cancelled for progression, death during neoadjuvant treatment due to Covid-19 infection, 1 lost to follow-up.*  *^#^ 1 surgery cancelled for progression, 1 surgery cancelled due to insufficient reduction of breast inflammation at the time of surgery, 3 lost to follow-up.* | | |

| **Supplementary Table 5** - Univariate analysis of the impact of demographic factors, occurrence of toxicities or treatment interruption on pCR. | | | | |
| --- | --- | --- | --- | --- |
|  | **N** | **pCR** | **OR (95%IC)** | **p-value** |
| **Age – years old**  ≥ 40  < 40 | 264  93 | 149 (56.4%)  55 (59.1%) | 1  1.12 (069-1.80) | 0.65 |
| **AJCC disease stage**  II  III | 262  95 | 149 (56.9%)  55 (57.9%) | 1  1.04 (0.65-1.68) | 0.86 |
| **TILs – %**  < 30  ≥ 30 | 224  126 | 118 (52.7%)  83 (65.9%) | 1  1.73 (1.10-2.73) | 0.017 |
| **Ki67 – %**  < 30  ≥ 30 | 30  327 | 6 (20.0%)  198 (60.6%) | 1  6.14 (2.44-15.43) | 0.0001 |
| **Androgen receptor expression**  Positive  Negative | 78  201 | 48 (61.5%)  108 (53.7%) | 1  0.73 (0.43-1.24) | 0.239 |
| **Treatment**  CT  CIT | 243  114 | 126 (51.6%)  78 (68.4%) | 1  2.01 (1.26-3.21) | 0.0034 |
| **Grade ≥ 3 AE**  No  Yes | 122  232 | 71 (58.2%)  131 (56.5%) | 1  0.94 (0.59-1.45) | 0.75 |
| **Reduction in dose**  No  Yes | 237  117 | 129 (54.4%)  73 (62.4%) | 1  1.39 (0.88-2.18) | 0.155 |
| **Postponement**  No  Yes | 215  139 | 121 (56.3%)  81 (58.3%) | 1  1.08 (0.7-1.67) | 0.71 |
| **Permanent discontinuation**  No  Yes | 279  75 | 162 (58.1%)  40 (53.3%) | 1  0.82 (0.49-1.38) | 0.46 |
| **Immune-related AE**  No  Yes | 50  63 | 38 (76.0%)  39 (61.9%) | 1  0.51 (0.22-1.17) | 0.12 |
| *AJCC: American Joint Committee on Cancer ; AE : adverse event ; CIT : chemoimmunotherapy ; CT : chemotherapy; OR : odds ratio ; pCR : pathological complete response ; TILs : tumor infiltrating lymphocytes ; 95%IC : 95% confidence interval.* | | | | |

| Supplementary Table 6 – Adjuvant treatments. | | | |
| --- | --- | --- | --- |
|  | **CT**  **N = 247** | **CIT**  **N = 119** | **p-value** |
| None | 130 (52.6%) | 14 (11.7%) | <0.000001 |
| Adjuvant treatment  Pembrolizumab  Capecitabine  Olaparib  Clinical trial  Other^%, €^ | 114 (46.2%)  -  94 (82.5%)  4 (3.5%)  12 (10.5%)  4 (3.5%)^%^ | 100 (84.0%)  72 (72.0%)  12 (12.0%)  1 (1.0%)  14 (14.0%)  1 (1.0%)^€^ |  |
| Radiotherapy, as per local guidelines | 238 (96.4%) | 111 (93.3%) |  |
| Missing data | 3 (1.2%) | 5 (4.2%) |  |
| *CIT : chemoimmunotherapy ; CT : chemotherapy.*  *^%^2 patients received adjuvant weekly Taxol, TAXOL hebdomadaire, 2 patients received endocrine therapy.*  *^€^1 patient received endocrine therapy* | | | |

| **Supplementary Table 7** – Details of outcomes according to neoadjuvant treatment*.* | | |
| --- | --- | --- |
|  | **CT**  **N = 247** | **CIT**  **N = 119** |
| **Follow-up** – median (month) | 38.7 | 18.9 |
| **Lost to follow-up** | 1 (0.4%) | 3 (2.5%) |
| **Event**  Progression during neoadjuvant treatment  Distant relapse  Other cancer  Death | 43 (17.4%)  6 (14.0%)  34 (79.1%)  2 (4.7%)*^&^*  1 (2.3%) | 8 (7.6%)  2 (25.0%)  5 (62.5%)  1 (12.5%)  - |
| **Relapse mode**  Locoregional  Controlateral  Metastases  Cerebral/meningeal  Thoracic  Abdominal  Non-visceral | 9 (25.7%)  3 (8.6%)  29 (82.9%)  9 (31.0%)  12 (41.4%)  9 (31.0%)  16 (55.2%) | -  -  5 (55.6%)  1 (20.0%)  2 (40.0%)  2 (40.0%)  2 (40.0%) |
| **Death**  Breast cancer  Adverse event during neoadjuvant treatment  Covid-19 infection  Pulmonary embolism | 18 (7.3%)  16 (88.9%)  -  1 (5.6%)  1 (5.6%) | 1 (0.8%)  1 (100%)  -  -  - |
| *CIT : chemoimmunotherapy ; CT : chemotherapy.*  *^&^1 kidney cancer, 1 HR+ breast cancer* | | |

| **Supplementary Table 8.** Summary of adverse events and pCR in the chemoimmunotherapy Institut Curie cohort and the KEYNOTE-522 study*.* | | |
| --- | --- | --- |
|  | **Institut Curie cohort** | **KEYNOTE-522** |
| **Treatement-related adverse events (non-immune) (%)** | | |
| Any grade | 100 | 98.9 |
| Grade ≥ 3 | 78.2 | 77.1 |
| **Immune-related adverse events (%)** | | |
| Any grade | 56.5 | 33.5 |
| Grade ≥ 3 | 11.3 | 12.9 |
| **Permanent discontinuation of neoadjuvant treatment due to toxicity (%)** | 31.1 | 27.7 |
| **pCR (%)** | 68.4 | 64.8 |
| *pCR : pathological complete response* | | |

**Supplementary Figure 1. Flow chart.**

Early triple negative breast cancer with neoadjuvant treatment indication

**N = 366**

Chemotherapy neoadjuvant treatment

(at least one dose received)

**N = 247**

Pembrolizumab-chemotherapy neoadjuvant treatment

*Between July 2021 and November 2022*

**N = 119**

2 canceled surgeries

*2 progressions*

1 lost to follow-up

1 death during neoadjuvant treatment due to Covid-19

2 canceled surgeries

*1 progression*

*1 insufficient reduction*

*of breast inflammation*

*before surgery*

3 lost to follow-up

1 lost to follow-up

2 treatments delegated to another hospital

3 lost to follow-up

1 treatment delegated to another hospital

Efficacy population

**N = 114**

Safety population

**N = 115**

Efficacy population

**N = 243**

Safety population

**N = 244**

**Supplementary Figure 2. Individual drug discontinuation according to treatment arm.**

**
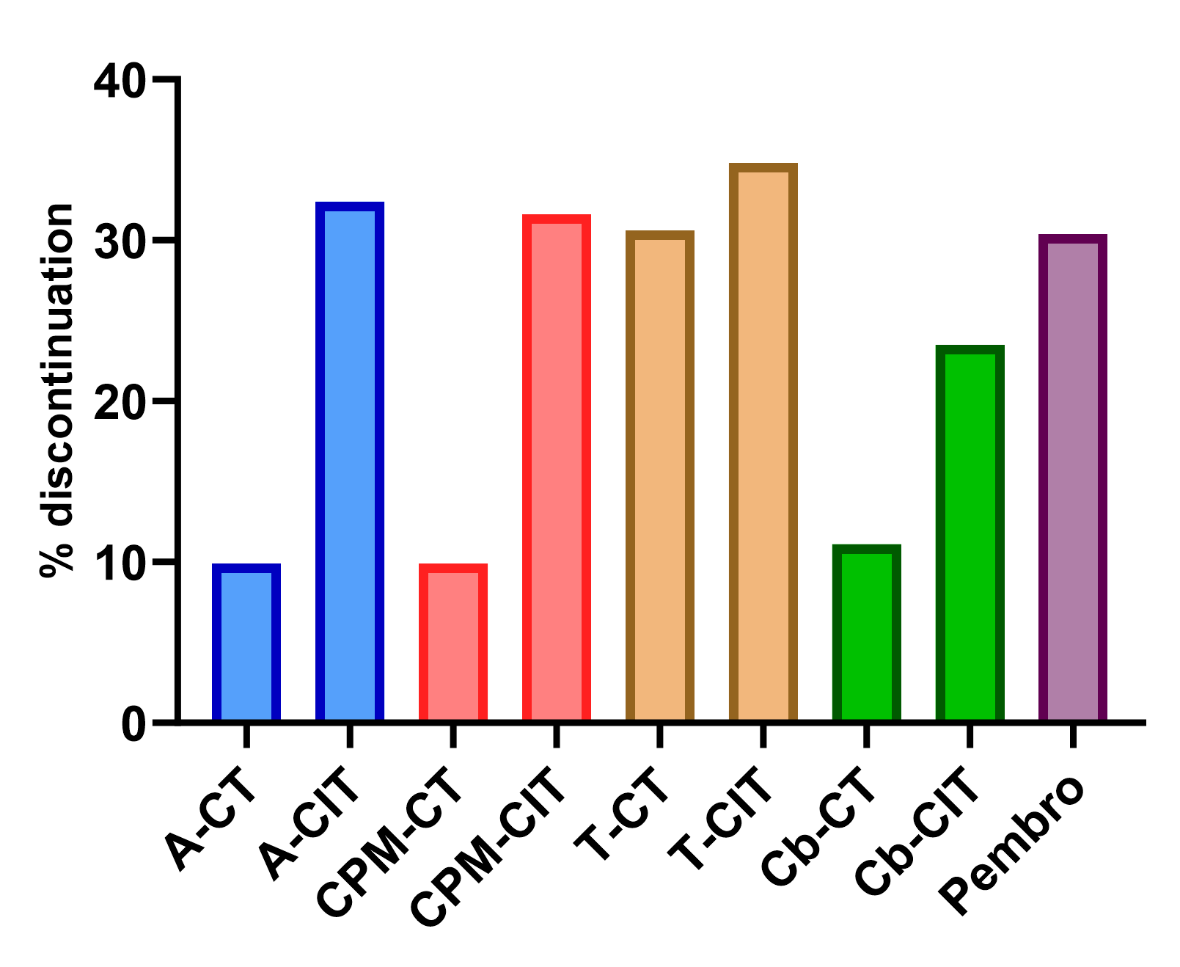
**

**Supplementary Figure 3. Relative dose intensity according to neoadjuvant treatment.**


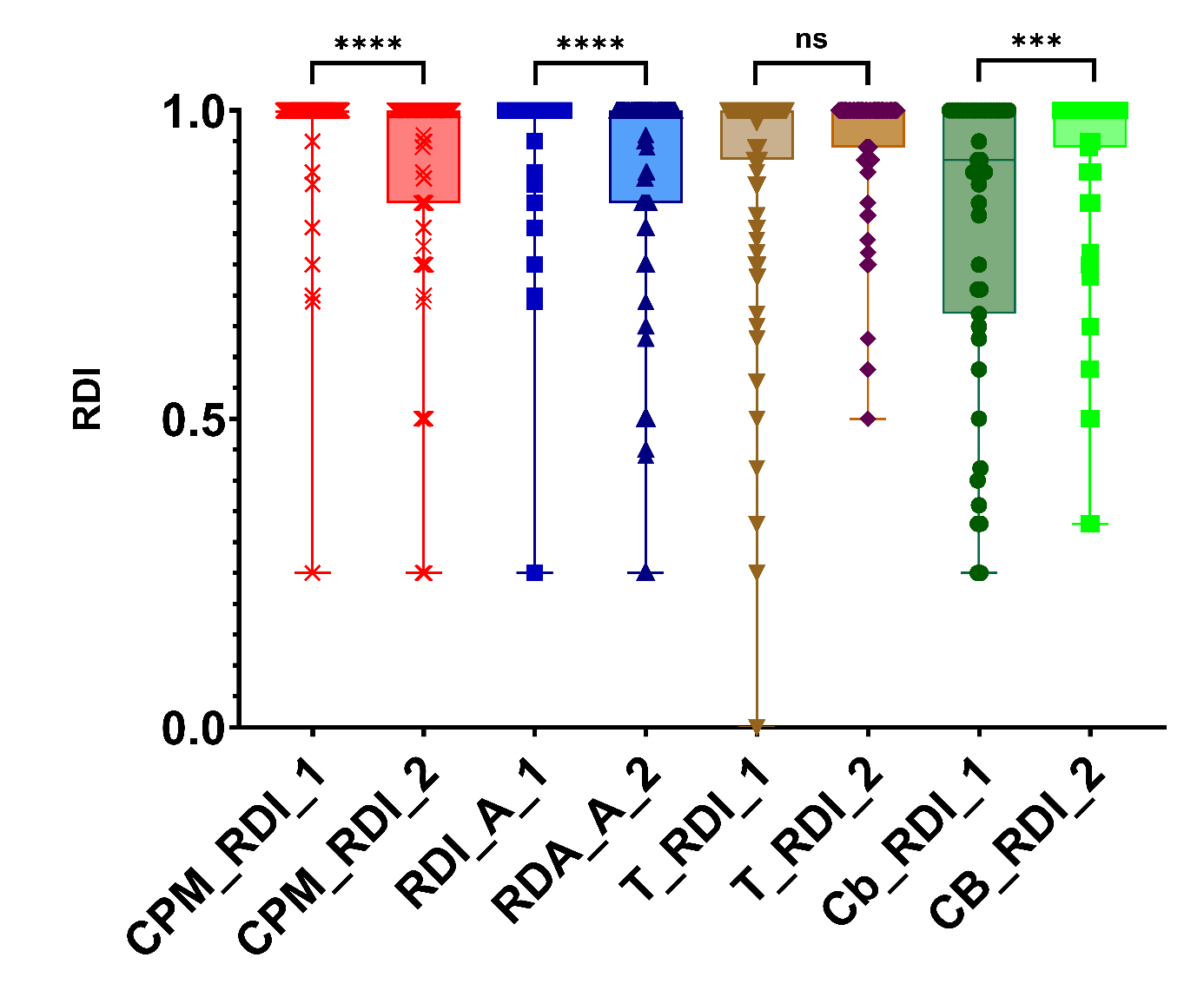


**Supplementary Figure 4. Outcomes according to pCR.**


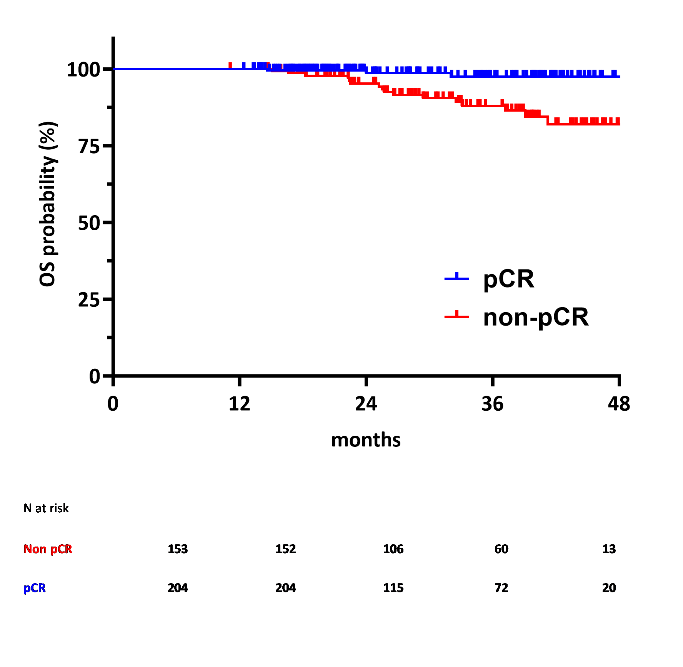

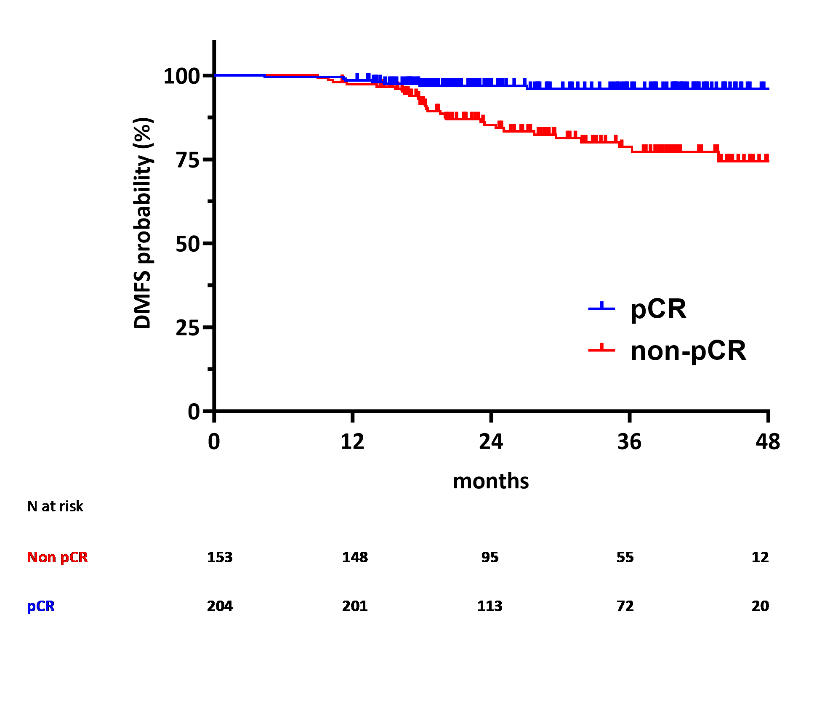

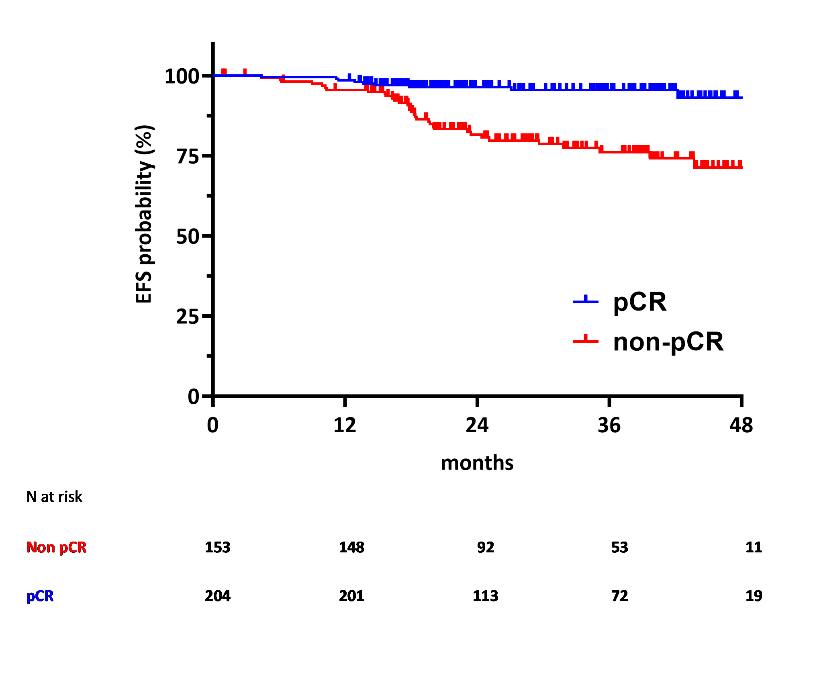

Supplement: Supplementary Data [file mmc1.docx]
